# Lessons Learned From the Battlefield and Applicability to Veterinary Medicine—Part 1: Hemorrhage Control

**DOI:** 10.3389/fvets.2020.571368

**Published:** 2021-01-14

**Authors:** Thomas H. Edwards, Michael A. Dubick, Lee Palmer, Anthony E. Pusateri

**Affiliations:** ^1^US Army Institute of Surgical Research, Joint Base San Antonio, San Antonio, TX, United States; ^2^Special Forces Group, Alabama Army National Guard, Auburn, AL, United States

**Keywords:** trauma, dogs, tourniquets, tranexamic acid, REBOA, hemorrhage, hemostatic dressing

## Abstract

In humans, the leading cause of potentially preventable death on the modern battlefield is undoubtedly exsanguination from massive hemorrhage. The US military and allied nations have devoted enormous effort to combat hemorrhagic shock and massive hemorrhage. This has yielded numerous advances designed to stop bleeding and save lives. The development of extremity, junctional and truncal tourniquets applied by first responders have saved countless lives both on the battlefield and in civilian settings. Additional devices such as resuscitative endovascular balloon occlusion of the aorta (REBOA) and intraperitoneal hemostatic foams show great promise to address control the most difficult forms (non-compressible) of hemorrhage. The development of next generation hemostatic dressings has reduced bleeding both in the prehospital setting as well as in the operating room. Furthermore, the research and fielding of antifibrinolytics such as tranexamic acid have shown incredible promise to ameliorate the effects of acute traumatic coagulopathy which has led to significant morbidity and mortality in service members. Advances from lessons learned on the battlefield have numerous potential parallels in veterinary medicine and these lessons are ripe for translation to veterinary medicine.

## Introduction

Since September 11, 2001, trauma care has advanced substantially, driven by the need to treat severely injured servicemen and women in the Iraq and Afghanistan wars. This has been possible due to military-civilian cooperation in trauma research, with significant advances resulting from work by both civilian and military researchers that have improved the care of the wounded and injured, both in and out of uniform (1–3). It is vital for the veterinary community to be aware of these advances in traumatology in order to evaluate the applicability of these advances in our patients.

Hemorrhage is the leading cause of potentially preventable death among military casualties (4) and the second leading cause among civilian trauma patients (5). Patients with trauma and severe hemorrhage require timely hemorrhage control combined with resuscitation to replace lost blood volume and mitigate the pathophysiologic consequences of hemorrhagic shock. Resuscitation will be addressed in a companion article. This article addresses advances in prehospital and emergency hemorrhage control, focusing primarily on non-surgical applications.

## Hemorrhage Control

Improved products and devices for hemorrhage control at the point of injury have been significant factors contributing to the wars in Iraq and Afghanistan having the lowest case fatality rate of any US war, despite increases in injury severity (6, 7). Major improvements in hemorrhage control that evolved over the course of these wars included the development of hemostatic dressings for treating externally compressible wounds, the re-introduction and technological advancement of limb tourniquets for extremity injuries, and devices such as junctional tourniquets for difficult to compress junctional wounds. Additionally, methods and devices have been developed that begin address pre-hospital treatment of intracavitary hemorrhage.

### Topical Hemostatic Dressings and Related Devices

**Figure 1:**
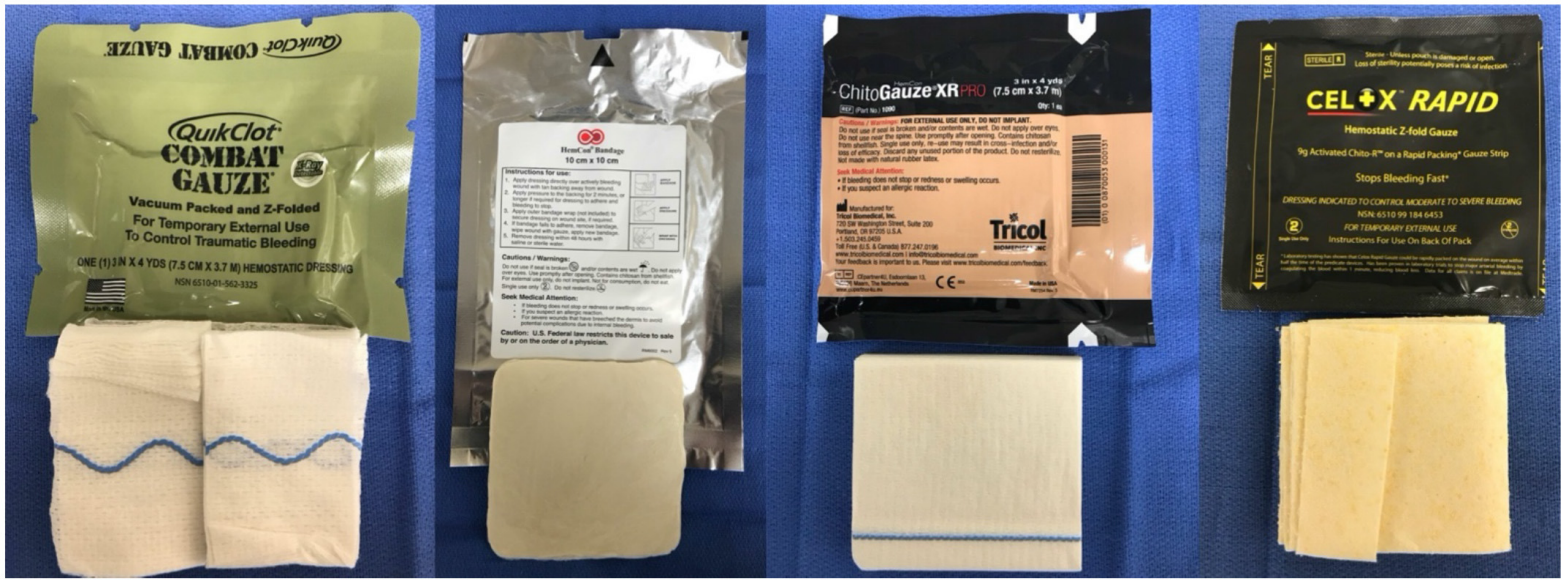
Four different hemostatic bandages that have been approved to stop moderate to severe hemorrhage.

In the late 1990's, focused efforts to develop topical hemostatic dressings that could control severe, life threatening bleeding in the prehospital environment were well-underway. After 9/11, efforts to find improved products intensified, with dressings being developed by many companies, universities, and others. The most promising dressings were ultimately tested in standardized models that challenged each dressing's ability to control severe venous (8) and severe arterial bleeding (9–13). These efforts resulted in the Committee on Tactical Combat Casualty Care (CoTCCC) recommending Combat Gauze®, a kaolin-coated gauze (Z-Medica, Wallingford, CT, USA), as the topical hemostatic dressing of choice for the US military. Based on additional research, this recommendation has now been expanded to include Celox Gauze (Medtrade Products Ltd, Crewe, UK) and ChitoGauze (Tricol Biomedical, formerly HemCon Medical Technologies, Portland, OR, USA), both chitosan—based dressings (14) (Figure 1). These dressings have been evaluated for up to 2–3 h in animal models, but longer term effectiveness remains unknown.

To specifically address deep penetrating wounds that may not be amenable to packing or direct compression, the XSTAT® (RevMedX, Wilsonville, OR, USA), was developed and received US Food and Drug Administration (FDA) clearance for the treatment of junctional and extremity bleeding. XSTAT® is made with expandable, cellulose mini-sponges which are delivered into a deep wound tract through a syringe-like applicator. Upon contact with blood, the non-absorbable sponges rapidly expand to fill the wound cavity, providing both a physical barrier and tamponade effect on the source of bleeding that facilitates coagulation (15, 16).

Another FDA-cleared hemorrhage control device is the iTClamp® (Innovative Trauma Care, Edmonton, Canada). The device seals the edges of the wound closed to produce temporary hemostasis. In both animal and human cadaver models, the iTClamp controlled bleeding from distal femoral, common femoral, carotid, and brachial artery injuries (17, 18).

Of note, none of the hemostatic dressings recommended by the CoTCCC or civilian EMS have shown significant efficacy against coagulopathic bleeding (14, 19). To date, fibrin dressings, which deliver both fibrinogen and thrombin, have been most effective in controlling coagulopathic bleeding (19, 20). A fibrin sealant patch (EVARREST®, Ethicon Inc., Somerville, NJ, USA) is FDA approved for soft tissue bleeding during open retroperitoneal, intra-abdominal, pelvic and non-cardiac thoracic surgery, but also appears effective in arterial and large venous bleeding (21–23). Due to cost and other factors, fibrin sealant dressings are primarily used in the surgical setting.

### Limb Tourniquets

As noted in several epidemiological assessments of casualties in past wars, extremities remain the most common site of injury in military conflicts, particularly today with the increased use of body armor (24). Although some type of tourniquet has been used for limb wounds throughout history, concerns over their safe use has been hotly debated (25, 26). Effective and easy to use tourniquets were developed and fielded by the military in the early 2000's. Since that time, several studies comparing casualties who were treated with tourniquets vs. those who were not, clearly demonstrated the life-saving value of this device to control extremity hemorrhage (27, 28). Additionally, a recent review of lower extremity arterial trauma in the military found no association between tourniquet use and limb loss (29). Currently, all deployed US Warfighters carry a tourniquet. There is also great potential for benefit in the civilian sector (30). In the past several years, tourniquet use has been incorporated by many civilian EMS, and over 500,000 people lay persons have been trained as part of the “Stop the Bleed” campaign (31). Examples of tourniquets currently approved for US Military use include the Special Operations Forces Tactical Tourniquet (SOFTT) and the Combat Application Tourniquet (CAT) (**Figure 4A**). Current efforts to develop or improve new or existing tourniquets are focused on making them easier to use and efforts continue to investigate pneumatic tourniquets to extend their application into pre-hospital situations.

### Junctional Tourniquets

Bleeding from junctional body regions accounts for about 20% of deaths from potentially survivable wounds in recent wars (4). These junctional body regions between the torso and its appendages (neck, groin, axilla, buttocks, pelvic area) are too proximal for a standard limb tourniquet to fit properly to control bleeding (32). An assessment of casualties from the recent wars in Iraq and Afghanistan found a large increase in junctional injury rates from 2001 to 2010 (33), and their recognition as a major cause of death from potentially survivable injuries led the US military to request junctional hemorrhage control devices as an urgent operational need in 2013 (34).

A number of devices were developed over a relatively short period through industry and military R&D efforts. Today, four junctional hemorrhage control devices are available: Combat Ready Clamp (CRoC) from CMS (Lafayette, NC, USA) the Junctional Emergency Treatment Tool (JETT; North American Rescue, Greer, SC, USA), SAM Junctional Tourniquet (SJT; SAM Medical Products, Wilsonville, OR, USA), and the Abdominal Aortic and Junctional Tourniquet (AAJT; Speer Operational Technologies, Greenville, SC, USA). At present the CRoC and SJT are cleared for groin and axillary bleeding, while the AAJT is cleared for groin, axilla and pelvic bleeding. The JETT is cleared only for groin hemorrhage at this time. It should also be mentioned that the SJT can also act as a pelvic splint and has US FDA clearance for stabilizing pelvic fractures. Although utilizing different mechanisms, junctional tourniquets are designed to apply pressure directly at the junctional region (e.g., femoral artery) or proximally (e.g., at the iliac artery or abdominal aorta via transabdominal application). These products have been tested in manikin, cadaver and animal studies, and there have been case reports in humans (35–39).

In a potential lethal femoral artery injury model in coagulopathic swine, the CRoC successfully stopped all hemorrhage and prevented rebleeding during fluid resuscitation; immediately after removing the clamp, bleeding ensued (40). While in-place, CT scans indicated no blood flow in the proximal, distal and collateral arteries of the clamped leg and only minor inflammation was observed after 1 h application of the clamp (40). A follow-on study examined the long-term effects of CRoC application. The clamp was applied for 2 h, followed by artery repair and reflow and recovery of the animals (41). Animals that received the CRoC recovered full mobility in the clamped leg within 9 days. Studies with the AAJT have reported its ability to reduce or eliminate blood flow at the common femoral artery, brachial artery and popliteal arteries in human volunteers. In an anesthetized swine model, the AAJT occluded the aorta and inferior vena cava for 60 min without bowel injury or significant potassium elevations, although others have observed metabolic acidosis and hyperkalemia after 1 h application in swine (42, 43). This latter study recommended ventilation of subjects upon release of the AAJT to improve survival. In clinical application, the AAJT was found effective in controlling bleeding from gunshot wounds to the axilla or left groin (28, 34). It was noted that in a casualty with traumatic bilateral amputations of the lower extremities, when the AAJT was placed around the torso as designed, it rapidly improved core physiological parameters (normal end-tidal CO_2_ and detection of a carotid pulse in this patient) (44). As may be expected, longer-term application is associated with more complications. Two-hour application at the level of the umbilicus in a swine model resulted in extensive muscle necrosis with functional disabilities (41).

### Intracavitary Hemorrhage

Although use of body armor has mitigated torso injuries, these injuries still occur, resulting in non-compressible intracavitary bleeding (45, 46). In fact, truncal, non-compressible hemorrhage accounted for about 67% of deaths from potentially survivable injuries in the recent military conflicts in Iraq and Afghanistan (47). Although significant progress has been made regarding all forms of hemorrhage that is externally compressible, non-compressible bleeding remains largely untreatable in the pre-hospital setting.

**Figure 2:**
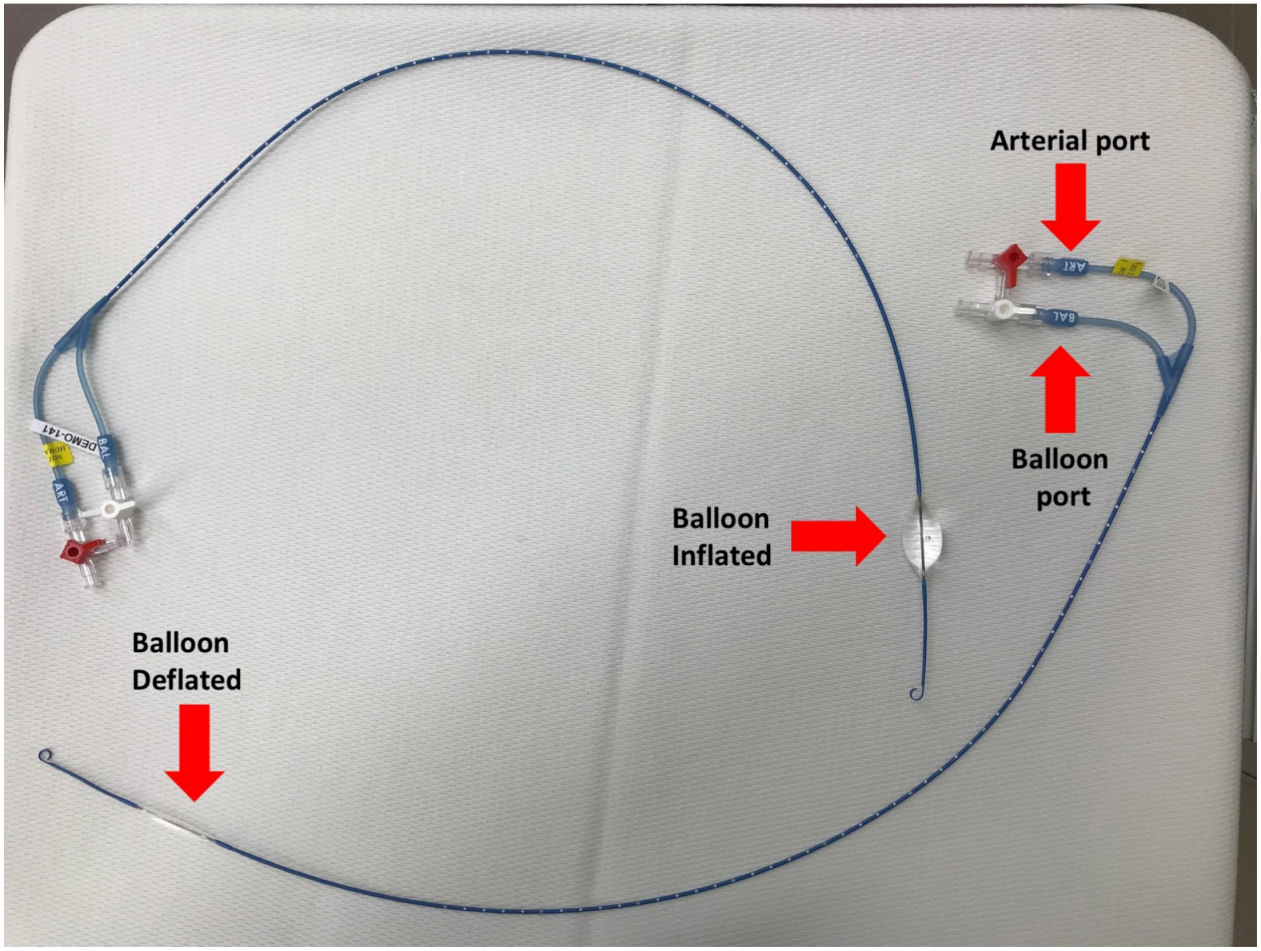
ER–REBOA–one with balloon inflated and one with balloon deflated.

One promising technique for achieving temporary control of intracavitary torso bleeding, as well as improving central blood pressure temporarily, is resuscitative endovascular balloon occlusion of the aorta (REBOA) (48). Although first reported to control non-compressible torso hemorrhage in the Korean Conflict, use of balloon occlusion of the aorta for hemorrhage control has seen a resurgence in recent years as an alternative to resuscitative thoracotomy (thoracotomy and aortic cross clamping). The goal of REBOA is to control hemorrhage temporarily to allow sufficient time for surgical repair of damaged organs and blood vessels associated with abdominal or other torso injuries. The ER-REBOA^TM^ catheter (Prytime Medical, Boerne, TX) allows guide-wire free application of REBOA without the need of advanced imaging, making it more applicable in emergency situations than previous devices that require advanced imaging (Figure 2). Current approaches involve insertion of the REBOA catheter via the femoral artery, advancing it to the level desired, and inflating the balloon to occlude distal blood flow (Figure 2). For the purposes of REBOA, the aorta is divided into the following levels or zones:

Zone I: extends from the origin of the left subclavian artery to the coeliac artery (~20 cm long in a young adult male)
Zone II: extends from the coeliac artery to the most caudal renal artery (~3 cm long)
Zone III: extends distally from the most caudal renal artery to the aortic bifurcation (~10 cm long).

An analysis of UK casualties in the Iraq and Afghanistan operations concluded that 1 in 5 had a hemorrhage focus in the abdomen or pelvic region that would be amenable to REBOA (49). Placement of REBOA in aortic zone I (balloon occlusion just cranial to the diaphragm) can raise blood pressure proximal to the balloon to support heart and brain circulation, while controlling arterial outflow to the disrupted distal circulation as demonstrated in a swine model (50, 51). Zone III inflation may be useful for pelvic bleeding in patients that are otherwise hemodynamically stable (51). Several pre-clinical feasibility studies have investigated REBOA for periods of 30 to 90 min in swine models with promising results, although inflammation (as demonstrated by a significant increase in IL-6 over baseline) and ischemic injury can be significant with prolonged occlusion times (50, 52–54). With clinical experience utilizing REBOA in humans vastly expounding, the American College of Surgeons and the American College of Emergency Physicians published a joint policy statement in support of emergency REBOA (55).

An expandable, polyurethane foam for intraperitoneal injection (Arsenal Medical, Watertown, MA, USA) is currently undergoing clinical evaluation for control of intra-abdominal hemorrhage (Arsenal Medical, REVIVE Trial, 2020). An initial evaluation in an uncontrolled liver injury in swine reported that the injected two liquid system polymerized into a foam in 2 min, expanded 30-fold in the abdomen and conformed to the internal organs, thereby, reducing blood loss and improving survival compared to untreated controls (56). Subsequent studies in this model or an iliac artery injury model reported similar efficacy results, but some bowel repair was necessary to assure long-term survival (57).

### Tranexamic Acid

Another substantial advance that has led to a significant increase in survival on the battlefield is the early administration of tranexamic acid (TXA). TXA is a lysine derivative that competitively inhibits the conversion of plasminogen to plasmin as well as plasmin activity; therefore, TXA prevents clot breakdown without inducing clot formation (58). It possesses a mechanism of action similar to that of ε-aminocarproic acid (EACA) but with ~10 times the potency (59). TXA was brought onto the frontline of trauma therapy after the publication of the landmark *Clinical Randomization of an Antifibrinolytic in Significant Hemorrhage-2* (CRASH-2) trial published in 2010 (60). With over 20,000 trauma patients enrolled, the CRASH-2 study showed a reduced all-cause mortality when TXA was given within 3 h of injury without an increased risk for thromboembolism; however, it demonstrated an increase in mortality if TXA was administered >3 h post-trauma. In a follow up study (*Military Application of Tranexamic acid in Trauma Emergency Resuscitation* or MATTERs) of nearly 900 severely injured service members, investigators found that TXA administration was associated with an increased survival benefit and less coagulopathy. This survival benefit was even more pronounced among the group that received a massive transfusion (61). Another large, prospective multicenter study investigated the use of TXA in people with acute traumatic brain injury. In this study, patients with mild to moderate traumatic brain injury who were given TXA showed a survival benefit. This survival benefit was most pronounced when the TXA was given soon after injury (62). The findings from these studies have led many military and civilian guidelines to strongly advocate for the early use of TXA in traumatized humans with hemorrhagic shock (63, 64). Using simulated models, Gayet-Ageron et al. demonstrated that the benefit of TXA decreased by 10% for every 15 min of treatment delay until the 3 h mark, after which there was no longer a benefit of TXA administration (65).

Recently however, some evidence has emerged which suggested that TXA may increase the incidence of thromboembolism in trauma patients. A retrospective evaluation of 455 military patients suffering trauma in Iraq and Afghanistan found an overall incidence of venous thromboembolism (VTE) of 15.6% and TXA was found to be an independent risk factor for VTE (66). Similarly, in a 2019 study, Myers and colleagues retrospectively evaluated almost 22,000 civilian trauma patients. They found a more than three-fold increase in odds of VTE in patients who received TXA (67). This recent data conflicts with previous reports which showed no significant increase in thromboembolic events in trauma patients administered TXA (60, 61). Further investigations will be necessary to elucidate the risk for VTE for human trauma patients administered TXA.

## The Veterinary Perspective

Prompt recognition and attenuation of life-threatening, trauma-induced hemorrhage are paramount to improve survival rates for the exsanguinating small animal patient. Similar to people, the main tenets for abating compressible hemorrhage in small animals are similar to those for humans and include direct digital pressure, pressure dressings, hemostatic agents and devices, and/or wound packing (68). The remainder of the article summarizes the translation of lessons-learned from human combat casualty care on the battlefield to that applicable for the veterinary general practitioner.

### Direct Pressure

Direct pressure applied to the source of bleeding is the most effective “medical” intervention for controlling most external hemorrhages, to include major arterial hemorrhages. Principles relative to direct pressure application in humans, also apply to canines with the following key concepts:

- Apply focal pressure directly over the source of bleeding at a force significant enough to staunch arterial flow.
- Maintain continuous pressure for a minimum of 5–10 min to allow initial thrombus formation.

**Figure 3:**
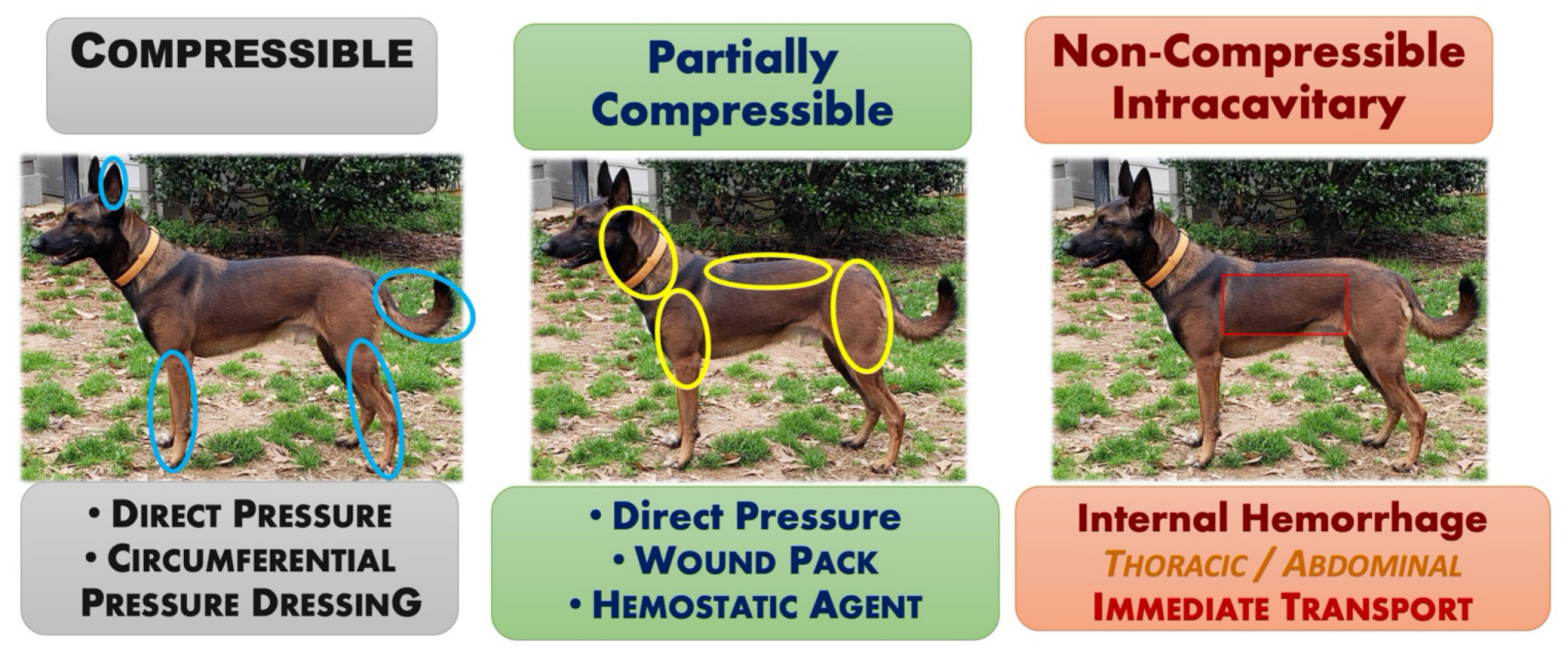
Compressible, partially and non-compressible areas of hemorrhage in a dog.

For small animals, the main methods of applying direct pressure predominantly include pressure bandages and wound packing. A pressure bandage is used to provide continuous pressure to a bleeding wound. Although, the pressure bandage provides pressure over the entire wound, applying more focal pressure directly over the main source of bleeding may increase its effectiveness. Where anatomically feasible, a *circumferential* pressure bandage achieves the greatest applied pressure for stopping arterial flow. *Non-circumferential* bandages often do not generate sufficient pressure, continuously, to successfully abate arterial hemorrhage. Wound packing refers to the technique of filling a bleeding, soft tissue injury or open wound with enough dressing or similar material to tamponade any bleeding vessels within the wound. For canines, this technique is most applicable for controlling deep compressible junctional hemorrhages and/or deep wounds in large muscle bellies in the neck, upper extremity above the elbow (triceps) and stifle (caudal thigh area), or in the perineal area (see Figure 3). Once the wound is packed a pressure dressing is placed over the top to maintain continuous pressure.

### Hemostatic Agents

In canines, evidence scientifically evaluating the application and effectiveness of hemostatic agents (kaolin and chitosan) and impregnated hemostatic dressings (QuikClot Combat Gauze, CELOX, ChitoGauze) described above is currently lacking. One study evaluated a chitosan dressing (Hemcon Bandages or Hemcon Patches) after femoral artery cannulation in 10 dogs. The author of this study found that the chitosan based dressing arrested bleeding in 12/14 arteries and the author concluded that the chitosan based dressings were generally successful in arresting bleeding from femoral arteries in dogs (69). Considering their demonstrated effectiveness in non-canine experimental animal models (porcine, ovine) along with anecdotal evidence from the field supporting the effectiveness of these agents in canines, the current opinion is that non-absorbable impregnated hemostatic dressings designed for humans are similarly considered effective in canines (12, 70). Granular type hemostatic agents (e.g., WoundStat®, TraumaCure, Inc.) are generally avoided due to their questionable efficacy for controlling major arterial hemorrhage along with their ability to complicate wound repair and cause an embolism to the brain and lungs (71).

### Hemostatic Devices

Similar to hemostatic agents, no studies currently exist specifically evaluating products such as the XSTAT or iTClamp. However, based upon their intended mechanism of action, in conjunction with demonstrated support from end-users, both products are considered effective for use in canines. Recommendation is to use the XSTAT or iTClamp similarly as they are licensed for in people: as an alternative to wound packing for controlling external hemorrhage from open and junctional wounds that are not amenable to tourniquet application. The XSTAT is not indicated for open wounds located into the thoracic or abdominal cavities.

### Limb Tourniquets

**Figure 4:**
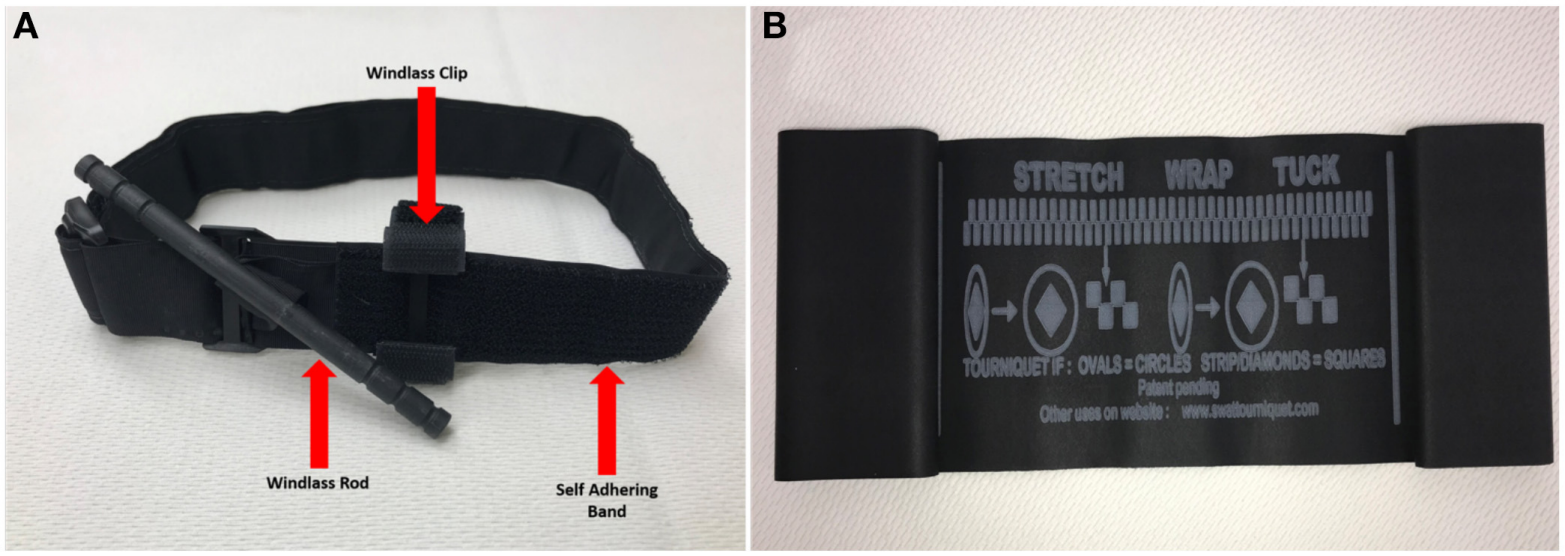
**(A)** Standard combat application tourniquet (CAT)—an example of a windlass tourniquet. **(B)** Stretch, wrap and tuck tourniquet (SWAT-T™).

**Figure 5:**
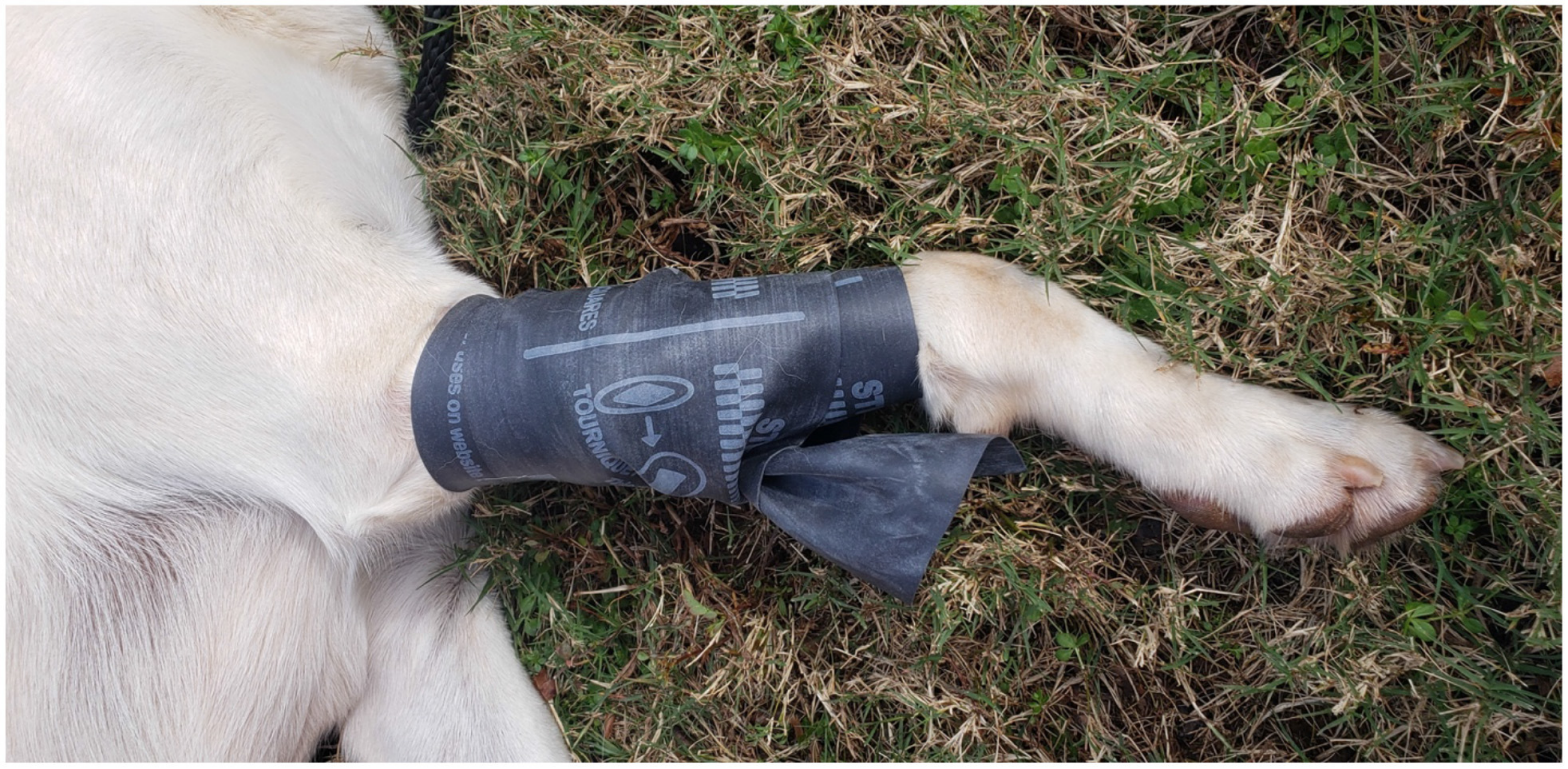
SWAT-T^TM^ tourniquet or pressure wrap on a canine forelimb.

Dissimilar to people, immediate tourniquet application for abating extremity hemorrhage is not as necessary of a life-saving intervention in canines as it is in humans. Evidence from the field along with the authors' professional experiences support that most extremity hemorrhages in canines do not warrant tourniquet application; instead, extremity hemorrhages, to include complete amputations, are immediately and effectively controlled with application of direct pressure and pressure bandages (72). Additionally, due to anatomical and conformational differences between humans and canines, commercial windlass tourniquets (C.A.T., SOFTT-W) designed for humans are ineffective. If able to tighten down adequately to occlude arterial flow, the tapered conformation of the canine's limb results in a greater incidence of tourniquet loosening and slippage; thus, resulting in only a brief and/or partial arterial occlusion (68). Recently, the stretchable, elastic “*Stretch, Wrap, And Tuck-Tourniquet*” or aka. *SWAT-T* (H&H Med Corp, Williamsburg VA) came on the market for use in people and canines (Figure 4B). The SWAT-T's stretchable and elastic nature allows it to “mold” to nearly any limb size and conformation (see Figure 5); therefore, eliminating the current problems encountered when attempting to apply a human-derived windlass tourniquet to a canine limb. The SWAT-T also serves as an effective pressure bandage, which is how it is primarily used for managing canine extremity hemorrhages. Currently, clinical field studies evaluating the use of the SWAT-T in canines are lacking.

### Junctional Tourniquets

Junctional tourniquets such as the AAJT, CRoC, SJT, and JETT have not been evaluated in canines and are not recommended at this time. Current recommendations for managing junctional hemorrhages include the aggressive application of direct pressure via wound packing, preferably using a hemostatic dressing to gain immediate hemostasis, followed by definitive surgical repair when staff and resources are available (73).

### REBOA

In veterinary medicine, no standardized clinical guidelines or evidence-based best practice protocols currently exist for using REBOA to manage non-compressible torso hemorrhage (74). In canines, available data regarding endovascular balloon occlusion remains limited mainly to experimental models, particularly, as it applies to CPR (74). It remains hopeful that the experiences and data gained from these experimental CPR models may, in some part, translate to clinical practice for managing non-compressible hemorrhage (51). Data from other non-CPR models using REBOA in canines are also currently available (75, 76). In a cadaver model, Loewen et al. demonstrated a 100% (15/15) success rate in placement of a REBOA catheter using the femoral artery in canines weighing 10 to 48 kg; the investigators used the 12th thoracic vertebrae as an external landmark for the catheter depth (76). Beyond the lack of scientific evidence and standardized clinical practice guidelines, current limitations for routinely implementing REBOA in small animal emergency trauma care include the availability and cost-prohibitive nature of REBOA resources (e.g., catheters) as well as the current lack of training regarding the advanced intravascular technique amongst most of the veterinary community. Additionally, performance of REBOA requires the staffing with the level of training and appropriate resources to provide critical patient care management while the catheter is in place, and then handle any complications (e.g., massive hemorrhage) that develop post-catheter removal.

### Antifibrinolytics

Results on antifibrinolytic therapy in small animal medicine has not been as straightforward as what has been shown in people. In a retrospective study examining the effectiveness of EACA in greyhounds who underwent amputation for appendicular tumors, dogs who did not receive EACA were 5.7 times more likely to bleed than the dogs who were given EACA (77). Another retrospective study, examining 122 dogs treated with EACA, revealed no correlation between the dose of EACA and blood administration, nor any correlation with the reason for hemorrhage. However, the study did suggest that EACA was well-tolerated in this group of dogs (78).

A number of studies have demonstrated that TXA inhibits fibrinolysis in dogs, both *in vivo* (79, 80) and *in vitro* (81, 82). Based on results of *in vitro* viscoelastic testing in the presence of tissue plasminogen activator, the dose to inhibit fibrinolysis was ~10-fold higher in canine than in human blood (82). It is not clear how this may relate to appropriate dosing in canines. In a study of 55 dogs who received TXA for intraoperative or post-operative hemorrhage, the group that received the TXA were treated with more plasma, but had a higher blood pressure and lower shock index than dogs who were not treated with TXA. Most importantly, dogs treated with TXA were less likely to be in shock at 24 h than those who were not treated with TXA (80). Kelmer et al. reported that transfusion volumes did not differ among dogs treated for clinical bleeding with TXA (83).

One reason for the apparent lack of (consistent) effectiveness of antifibrinolytics in reducing clinical bleeding in these studies may have been small sample sizes studied, considering that the trials that demonstrated efficacy in humans have included thousands of patients. More clinical studies funded by the Department of Defense are currently underway looking at TXA use in naturally injured dogs and the results of these studies will hopefully shed light into the appropriate use of TXA.

## Conclusion

In summary, several advances in hemorrhage control have been realized since the US has been involved in recent wars, including hemostatic dressings and devices, limb and junctional tourniquets, and new devices and procedures to address non-compressible torso hemorrhage. Judicious use of large animal models, primarily swine, have allowed these techniques and devices to result in human lives saved after traumatic injury. Currently, the long term effectiveness of hemostatic dressings are unknown, and safety concerns regarding long-term application of the hemostatic devices mentioned significantly limit their application under prolonged care scenarios. Nevertheless, many of these advances are ripe for incorporation into general and specialty practices where veterinarians are treating small animals with significant traumatic injuries.

## Author Contributions

TE, MD, and LP drafted the manuscript. TE, MD, LP, and AP reviewed and edited the manuscript. TE and AP obtained funding. All authors contributed to the article and approved the submitted version.

## Conflict of Interest

The authors declare that the research was conducted in the absence of any commercial or financial relationships that could be construed as a potential conflict of interest. The handling editor declared a shared committee with one of the authors LP at the time of review.
